# Appendicoumbilical Fistula: A Rare Reason for Neonatal Umbilical Mass

**DOI:** 10.1155/2011/835474

**Published:** 2011-05-02

**Authors:** M. Cevik, M. E. Boleken, E. Kadıoglu

**Affiliations:** ^1^Department of Pediatric Surgery, Faculty of Medicine, Harran University, 63100 Sanliurfa, Turkey; ^2^Department of Pathology, Sanliurfa Education and Training Hospital, Sanliurfa, Turkey

## Abstract

The normal umbilicus is a simple structure, but the intrauterine development of the umbilicus is highly complex. Neonatal umbilical mass anomalies usually represent failure of obliteration of the vitelline duct or the allantois which results in persistence of remnants, which can lead to a wide variety of disorders. In this paper, we present a case of an appendicoumbilical fistula in a neonate along with the differential diagnosis and management options. Embryologic explanation of the etiology was discussed with the possible association with different forms of malpositioning and rotation of the gut.

## 1. Introduction

The differential diagnosis of an umbilical mass in the neonatal period includes persistent omphalomesenteric (vitelline) duct, herniated Meckel's diverticulum, urachal cyst, and umbilical polyp. In a previous paper, embryonic remnants were found in 231 cases (23.1%), which included remnants of the allantoic duct (63%), omphalomesenteric duct (6.6%), and embryonic vessels (30.4%) [1].

In this paper a case of a neonatal appendicoumbilical fistula will be presented, and presentation, differential diagnosis, and etiology will be discussed.

## 2. Case Report

A full-term 2-day-old male was referred to our clinic for evaluation of an umbilical mass. Umbilical cord was normal except that there was a mass on its edge. The mass was 3 cm in diameter, fixed and erythematous with a fistulous opening on its top. Abdominal ultrasound was normal. A fistulogram that was done through the fistulous opening revealed an irregular track extending to cecum.

The child underwent emergent umbilical stump exploration. The skin was incised subumbilicaly circumferentially. Umbilical arteries and vein were identified. The umbilical vessels were ligated with silk ties. The mass was connected to the 1/3 proksimolateral appendix. Cecum was mobile, and cecal vessels were abnormal. After ligation of the appropriate mesenteric vessels, appendectomy along with the fistula tract and mass was done. Final pathologic analysis revealed a normal appendix and mesoappendix.

## 3. Discussion

Reports of a fistulous connection between appendix and umbilicus are rare. Collins [2] reported only three cases in a review of 50000 specimens of the human vermiform appendix. He did not describe the etiology as congenital. This makes it a difficult event to study and keeps its pathophysiology and management unclear. We believe that in our case the fistula is congenital because of the early presentation without any history of infection or trauma and the finding of mobile cecum during exploration. 

With normal developmental anatomy by the fifth week of gestation the vitelline duct forms the connection between the apex of the midgut (primary inte,stinal duct) and the yolk sac. Around the sixth week, the midgut starts to grow rapidly, and the increasing intra-abdominal pressure forces the midgut to protrude and enter the umbilical cord. The midgut then differentiates into jejunum, ileum, ascending colon, and transverse colon, while at the same time the appendix sprouts from the cecum. By the tenth week, the vitelline duct detaches from the ileum and becomes obliterated. The herniated loop of the midgut starts to retract and re-enters the abdominal cavity. This process is completed by the 11th week [3]. Delay of involution of the vitelline duct has been suggested to hinder the complete return of the midgut by a Meckel's diverticulum adherent to the apex of small exomphalos sac. A similar mechanism may be responsible for the formation of an appendicoumbilical fistula. Nonrotation or malrotation of bowel associated with appendicoumbilical fistula and also aberrant blood supply of the appendix being found anterior to the cecum further supports this etiology [4]. 

Our patient that was presented with an umbilical mass is the second appendicoumbilical fistula case with a preoperative diagnosis in the literature [5]. He had a normal umbilical cord with irreducible, erythematous intestinal segment on its side. The differential diagnosis upon examination of this patient in clinic included incarcerated hernia of Meckel's diverticulum or a persistent urachal remnant. Abdominal USG excluded those possibilities. USG in the hands of an excellent sonographer should be a gold standard, which is turned today towards minimizing childhood radiation in early childhood, but it is not easy to find a radiologist who has the hands of an excellent sonographer. Fistulogram delineated a track which was ending towards right iliac fossa without the hands of a radiologist. Thus, fistulogram is still the gold standard for diagnosis. Umbilical exploration should be done as soon as possible in such cases for the fear of having volvulus around the fistula tract because the fixation of the bowel may be deficient.

## References

[1] Jauniaux E, De Munter C, Vanesse M, Wilkin P, Hustin J (1989). Embryonic remnants of the umbilical cord: morphologic and clinical aspects. *Human Pathology*.

[2] Collins DC (1955). A study of 50,000 specimens of the human vermiform appendix. *Surgery, Gynecology and Obstetrics*.

[3] Larsen W (1997). *Human Embryology*.

[4] Brogna-Pignatti C, Bergamo Andreis I, Betili G, Zamboni G (1995). Delayed separation of an appendix-containig umbilical stump. *Journal of Pediatric Surgery*.

[5] Pal K, Singh VP (2006). Appendico-umbilical fistula. *Indian Pediatrics*.

